# Schmiedeberg Medal for Thomas Wieland: an artist of G-protein signaling

**DOI:** 10.1007/s00210-025-04101-2

**Published:** 2025-04-07

**Authors:** Susanne Lutz, Friederike Cuello, Roland Seifert

**Affiliations:** 1 Institute of Pharmacology and Toxicology, University Medical Center Göttingen, Robert-Koch-Str. 40, 37075 Göttingen, Germany; 2 Institute of Experimental Pharmacology and Toxicology, University Medical Center Hamburg-Eppendorf, Hamburg, Germany; 3 Institute of Pharmacology, Hannover Medical School, Hannover, Germany

**Figure:**
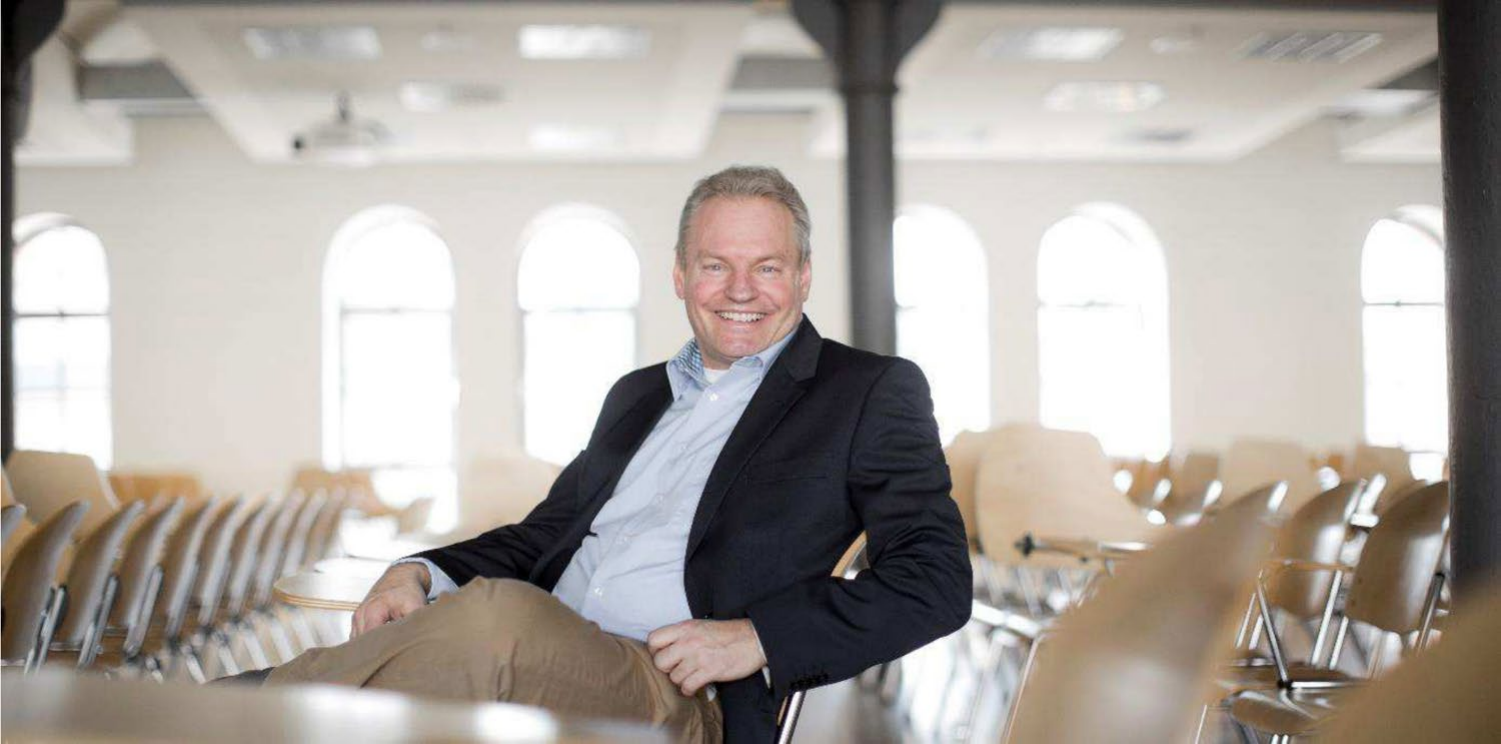
© Alexander Grüber / University Medical Center Mannheim (UMM)

We, the authors, are honored to announce that the German Society for Experimental and Clinical Pharmacology and Toxicology (DGPT) has awarded the Schmiedeberg Medal to Professor Thomas Wieland, a pharmacologist at heart and a role model of the highest order.

The Schmiedeberg Medal is the highest honor bestowed by the DGPT and is awarded to scientists who have made continuous outstanding contributions to the field of pharmacology and toxicology over decades. In this tradition, Thomas Wieland is a worthy successor to the previous honorees.

Thomas Wieland studied pharmacy in Heidelberg and received his PhD from the Institute of Pharmacology at the University of Heidelberg under the supervision of Prof. Karl-Heinz Jakobs, who was the first to postulate the existence of inhibitory G-proteins (Jakobs et al. 1979). It was during this time that Thomas Wieland became chronically infected with G-protein research, which he has pursued with great success ever since. Remarkably, already in Thomas Wieland’s first publication as a co-author and young PhD student in 1988, he and his colleagues laid the foundation for his passion for his still favorite protein, the nucleoside diphosphate kinase (NDPK) (Seifert et al. 1988).

Several publications later and after receiving his Ph.D. in 1989, Thomas Wieland remained in Heidelberg as a postdoctoral researcher in the group of Prof. Jakobs and moved with him to Essen as a junior group leader in 1991. During this time, Thomas Wieland met his second scientific love besides NDPK (Wieland et al. 1991), the monomeric G-proteins (Wieland et al. 1990a, b), and he worked eagerly on several other questions related to G-protein signaling. Together with his esteemed mentor, he published 14 first- or last-author papers and 8 co-author papers between 1990 and 1996, before leaving Germany for a research stay at the prestigious California Institute of Technology. There, Thomas Wieland worked for a year and a half in the research group of Prof. Melvin I. Simon, himself a renowned scientist in the field of G-proteins. Here Thomas Wieland first came into contact with a new class of G protein regulators, the RGS (regulator of G-protein signaling) family of proteins (Chen et al. 1996; Wieland et al. 1997, 2000).

After a very successful stay abroad and his habilitation in the subject Pharmacology and Toxicology at the University of Duisburg-Essen in 1996, Thomas Wieland was appointed Full Professor of Pharmacology at the Institute of Experimental Pharmacology and Toxicology at the University Medical Center Hamburg-Eppendorf in 1997. In 2002, he was appointed Professor at the Institute of Pharmacology and Toxicology at the Mannheim Medical Faculty of the University of Heidelberg, and in 2007, he was appointed Director of this Institute. In 2018, the Institute became part of the newly founded European Center for Angioscience and Thomas Wieland became head of the Department of Experimental Pharmacology.

Throughout his career, Thomas Wieland has actively shaped the field of G-proteins in general and their role in the cardiovascular system in particular. His endogenous curiosity and sense for scientific puzzles, combined with his understanding of collaborative research, has led him to publish 182 papers until now in highly regarded journals, including Science, Circulation, and Proceedings of the National Academy of Sciences. He realized this remarkable effort with numerous collaborators, most of whom have worked with him repeatedly, demonstrating the scientific community’s appreciation of Thomas Wieland as a scientist and friend.

To better understand Thomas Wieland as a scientist, a brief summary of his contribution to the understanding of NDPK in cardiac physiology and pathology may be helpful. In the past, many researchers considered NDPK to be a housekeeping enzyme that catalyzes an unspectacular “ping-pong” phosphate transfer from ATP to GDP and thus contributes to G-protein signaling simply by keeping the GTP pool in cells high. Thomas Wieland has shifted this perception and demonstrated that this is far from the truth. In persistent hard work, he has evolved the role of this kinase from a mere phosphorylating entity that could potentially transfer a thiophosphoryl group to endogenous GDP (Seifert et al. 1988) to a hexameric, heterogeneous signaling molecule that transduces its signal by phosphorylating Gβ, that regulates the stability and localization of heterotrimeric G-proteins, contributes to cardiomyocyte contractility, and is involved in dysregulated signaling in human heart failure (Wieland et al. 2000; Cuello et al. 2003; Hippe et al. 2009, 2011; Abu-Taha et al. 2017). In addition, Thomas Wieland and his group have extended the role of NDPK for endothelial integrity (Shan et al. 2018; Chatterjee et al. 2020a, b). Taken together, Thomas Wieland’s work has demonstrated that the NDPK is a worthy drug target for several cardiovascular diseases. His collaborative work also suggests a role for NDPK in cancer cell survival and metastasis (Trova et al. 2023; Ferrucci et al. 2024). In his most recent study, he laid the groundwork for NDPK-targeted drug development by demonstrating the resolved structure of the NDPK C isoform, which is critical for regulating G-protein signaling in heart failure (Abu-Taha et al. 2017). Thomas Wieland’s passion for the NDPK makes him a highly respected scientist in the NDPK community, and he is certainly very active in various leading roles in the organization of the International Conference on the NME/NDPK/NM23/AWD Gene Family, which takes place every three years at a different location. The conference was organized by Thomas Wieland in Mannheim in 2010.

Thomas Wieland’s ability to juggle signaling proteins like an artist in the circus arena becomes even more apparent when one considers his work in the field of monomeric G-proteins. Where other scientists might struggle with almost linear signaling cascades, Thomas Wieland thrives when it comes to more complex, interconnected pathways. He has demonstrated his mastery in his diverse work at the interface between heterorimeric and monomeric G-proteins. Exemplary for his extensive contribution to the field and in a very simple description, he showed among others that G_q/11_ proteins are connected to RhoA by the Rho guanine nucleotide exchange factor p63RhoGEF (Lutz et al. 2007; Wuertz et al. 2010) and established a role for RGS3L as a switch factor between Rac1 and RhoA signaling (Vogt et al. 2007; Levay et al. 2022).

Thomas Wieland has received several awards, including the 2003 Albert Fraenkel Prize, which is awarded by the German Society of Cardiology (DGK) to German scientists who have distinguished themselves through publications in the field of physiology, pharmacology, pathology, clinic, or therapy of the circulatory system. Furthermore, he was awarded by the DGK in 2016 at the 82. Annual Meeting of the DGK the “Honory Award Lecture on Basic Science.” In 2011, Thomas Wieland received the EFSD/Sanofi-Aventis European Research Award in Micro- and Macrovascular Complications of Diabetes.

Thomas Wieland’s careful and inspiring way of mentoring young researchers may have contributed to his scientific success. With his curiosity and vast knowledge, he imbued many young researchers with his ideas about the importance of NDPK, monomeric G-proteins, and accessory G-protein regulators in cell biology and various human diseases, and made them work hard with enthusiasm, even if it meant repeatedly isolating transducin from fresh cow eyes obtained from the slaughterhouse in a freezing dark cold room with red-light as the only light source for hours.

For us, the authors, it is also important to state that Thomas Wieland never made a big deal about gender equality in science, nor did he force female scientists to participate in well-intentioned but sometimes questionable special training programs. Instead, Thomas Wieland unreservedly supports and encourages female scientists in their daily research lives and, more importantly, in the advancement of their scientific careers. In fact, his advice is highly valued by many of the female scientists in the field, who seek his counsel on a regular basis. As a result, up to now, two of his female mentees were able to obtain professorships in pharmacology, which, as far as we know, remains unique in Germany to date.

In addition to his commitment to all aspects of pharmacological research, Thomas Wieland is a dedicated teacher and has a keen sense of the future challenges in science and those facing our healthcare system. Between 2005 and 2008, Thomas Wieland was instrumental in structuring the training program for the German “Fachpharmakologe/Fachpharmakologin DGPT.” This program serves to train and certify scientists with a background in the life sciences in the field of pharmacology. Long considered unimportant by many pharmacologists, this program fulfills all the criteria for the training of so-called medical scientists, which today, and thus remarkably 20 years later, is on everyone’s lips (Streckfuss-Bomeke et al. 2024).

In 2015, Thomas Wieland was appointed Dean of Studies at the Mannheim Medical Faculty of the University of Heidelberg. And as one would expect from someone as energetic as Thomas Wieland, he has dedicated himself to improving the education of medical students and the long-term perspective of medical care in Germany. Two representative, outstanding and innovative projects, that have been implemented under his leadership as Dean of Studies should be mentioned here. First, the initiation of the Mannheim Interprofessional Training Ward (MIA) in 2017, in which students from different disciplines are trained together, and the AMBIGOAL research project, which aims to create innovative health centers in rural areas and is funded by the state of Baden-Württemberg.

But Thomas Wieland is not only an outstanding pharmacological scientist, a mentor, and a gifted teacher, he is also deeply committed to giving pharmacology the reputation it deserves. He has held and continues to hold various positions in scientific societies such as the DGPT, the European Pharmacological Society (EPHAR), and the DGK, in which he fulfills his duties with integrity and diplomacy and never for personal gain.

It would go beyond the scope of this laudation to describe all of Thomas Wieland’s positions and achievements in scientific societies and consortia, so only a few will be mentioned here. Thomas Wieland was a member of several committees of the DGK; currently, he is a member of the Committee for Scientific Quality. He is a principal investigator of the German Center for Cardiovascular Research (DZHK) at the Heidelberg/Mannheim site and was co-spokesperson from 2017 to 2019. Importantly, Thomas Wieland has shaped the DGPT in his role as Chairman of the DGP Board from 2017 to 2023 and as President of the DGPT in 2022. He also hosted the 2nd German Pharm-Tox Summit in Heidelberg in 2017. His outstanding commitment as a pharmacologist within the German Research Foundation (DFG) should also be mentioned, where he served as a member of the Review Board for Pharmacology and spokesperson of the Review Board Medicine 3 from 2017 to 2024. In this role, he succeeded in increasing the number of members of the Pharmacology Review Board, which is essential for all scientifically active pharmacologists.

Today, Thomas Wieland shows his foresight as a European and is very active in his position as a committee member of the Federation of European Pharmacological Societies (EPHAR) since 2022 to connect German pharmacology with its European family.

We, the authors, know that the achievements of Thomas Wieland described here are far from complete, and we apologize that we have not been able to honor his many and important contributions to pharmacology by not mentioning them in this laudation. We hope, however, that this laudation is sufficient to express our deepest appreciation for Thomas Wieland, a pharmacologist at heart and a role model of the highest order.

It is a great honor for the DGPT to award the Schmiedeberg Medal to Professor Thomas Wieland.

## Data Availability

All source data for this work (or generated in this study) are available upon reasonable request.
